# Predictors of future suicide attempt among adolescents with suicidal thoughts or non-suicidal self-harm: a population-based birth cohort study

**DOI:** 10.1016/S2215-0366(19)30030-6

**Published:** 2019-04

**Authors:** Becky Mars, Jon Heron, E David Klonsky, Paul Moran, Rory C O'Connor, Kate Tilling, Paul Wilkinson, David Gunnell

**Affiliations:** a Population Health Sciences, University of Bristol Medical School, Bristol, UK; b National Institute for Health Research Biomedical Research Centre, University Hospitals Bristol NHS Foundation Trust, University of Bristol, Bristol, UK; c Department of Psychology, University of British Columbia, Vancouver, BC, Canada; d Suicidal Behaviour Research Laboratory, Institute of Health and Wellbeing, University of Glasgow, Glasgow, UK; e University of Cambridge and Cambridgeshire and Peterborough NHS Foundation Trust, Cambridge, UK

## Abstract

**Background:**

Suicidal thoughts and non-suicidal self-harm are common in adolescents and are strongly associated with suicide attempts. We aimed to identify predictors of future suicide attempts in these high-risk groups.

**Methods:**

Participants were from the Avon Longitudinal Study of Parents and Children, a population-based birth cohort study in the UK. The sample included 456 adolescents who reported suicidal thoughts and 569 who reported non-suicidal self-harm at 16 years of age. Logistic regression analyses were used to explore associations between a wide range of prospectively recorded risk factors and future suicide attempts, assessed at the age of 21 years.

**Findings:**

38 (12%) of 310 participants with suicidal thoughts and 46 (12%) of 380 participants who had engaged in non-suicidal self-harm reported having attempted suicide for the first time by the follow-up at 21 years of age. Among participants with suicidal thoughts, the strongest predictors of transition to attempts were non-suicidal self-harm (odds ratio [OR] 2·78, 95% CI 1·35–5·74; p=0·0059), cannabis use (2·61, 1·11–6·14; p=0·029), other illicit drug use (2·47, 1·02–5·96; p=0·045), exposure to self-harm (family 2·03, 0·93–4·44, p=0·076; friend 1·85, 0·93–3·69, p=0·081), and higher levels of the personality type intellect/openness (1·62, 1·06–2·46; p=0·025). Among participants with non-suicidal self-harm at baseline, the strongest predictors were cannabis use (OR 2·14, 95% CI 1·04–4·41; p=0·038), other illicit drug use (2·17, 1·10–4·27; p=0·025), sleep problems (waking in the night 1·91, 0·95–3·84, p=0·069; insufficient sleep 1·97, 1·02–3·81, p=0·043), and lower levels of the personality type extraversion (0·71, 0·49–1·03; p=0·068).

**Interpretation:**

Most adolescents who think about suicide or engage in non-suicidal self-harm will not make an attempt on their life. Many commonly cited risk factors were not associated with transition to suicide attempt among these high-risk groups. Our findings suggest that asking about substance use, non-suicidal self-harm, sleep, personality traits, and exposure to self-harm could inform risk assessments, and might help clinicians to identify which adolescents are at greatest risk of attempting suicide in the future.

**Funding:**

American Foundation for Suicide Prevention, National Institute for Health Research Biomedical Research Centre at the University Hospitals Bristol National Health Service Foundation Trust, and the University of Bristol.

## Introduction

Suicidal behaviour is a major public health concern in adolescents. Although suicidal thoughts and non-suicidal self-harm are strong predictors of suicide attempts, little is known about the factors that predict attempts in these high-risk groups. A better understanding of these factors is crucial for improved suicide prediction and prevention.

Only a third of adolescents who have suicidal thoughts are estimated to go on to make a suicide attempt.^1^ Theoretical models of suicide, including the interpersonal theory,^2^ the integrated motivational–volitional model,^3^ and the three-step theory,^4^ are consistent with an ideation-to-action framework. This framework proposes that the factors involved in the development of suicidal thoughts are distinct from those involved in the transition from thoughts to attempts. Several large epidemiological and meta-analytical studies provide empirical support for this framework and have found that many well established risk factors for suicide (such as depression, impulsivity, and hopelessness) do not meaningfully differentiate individuals with suicidal thoughts from those who have made an attempt.^1^, ^5^, ^6^, ^7^ According to a recent review,^8^ the factors that most consistently predict suicide attempts among people with ideation relate to suicide capability (ie, the degree to which an individual feels able to make a suicide attempt). In a previous study of more than 4500 adolescents,^9^ we explored a wide range of risk factors and found that exposure to self-harm in others, psychiatric disorders, and substance use most strongly distinguished between adolescents with suicidal thoughts and those who acted on those thoughts. However, like most previous studies exploring this issue,^1^, ^10^, ^11^, ^12^, ^13^, ^14^ the analyses were cross-sectional, and the extent to which these factors would predict future suicide attempts is currently unknown.

Much of the scientific literature and theory exploring transitions to suicide attempts has focused on suicidal thoughts. However, investigation of predictors of attempts among people who engage in non-suicidal self-harm is also important, because this factor is strongly associated with suicide attempt history and predicts future attempts in longitudinal studies.^15^, ^16^, ^17^, ^18^, ^19^ A meta-analysis of 52 studies (all using retrospective self-report) found that the strongest correlates of suicide attempts among adolescents who engaged in non-suicidal self-harm were suicidal ideation, hopelessness, and non-suicidal self-harm characteristics (frequency and number of methods).^20^ As found for suicidal thoughts, many often-cited risk factors for suicide were generally poor at distinguishing between adolescents with suicidal and non-suicidal self-harm. The only previous longitudinal study^21^ also found self-harm frequency to be an important predictor of suicidal behaviour among adolescents who engage in non-suicidal self-harm. Other factors identified were reduced social connectedness and sense of meaning in life, and increased levels of mental health treatment.

**Research in context**

**Evidence before this study**

Suicidal thoughts and non-suicidal self-harm are strongly associated with suicide attempts. However, the majority of adolescents who think about suicide or engage in non-suicidal self-harm will not make an attempt on their life. We searched PubMed for studies published in English before Dec 13, 2018, investigating risk factors for suicide attempts among these high-risk groups. We did two separate searches of the scientific literature. One search was for suicidal thoughts using the query (“suicidal thoughts” OR “suicidal ideation”) AND (“suicide attempt” OR “suicidal behaviour” OR “ideation to action”). The other search was for non-suicidal self-harm using the query (“non-suicidal self-harm” OR “non-suicidal self-injury” OR “NSSI”) AND (“suicide attempt” OR “suicidal behaviour”). We also checked citations of relevant publications and searched the reference lists of selected articles. Existing research suggests that many well established risk factors for suicide (such as depression, hopelessness, and impulsivity) do not predict suicide attempts among adolescents who have suicidal thoughts or engage in non-suicidal self-harm. Longitudinal studies investigating predictors of future suicide attempts in these high-risk groups are extremely scarce.

**Added value of this study**

This is the first population-based birth cohort study to explore predictors of future suicide attempts among adolescents who have suicidal thoughts or engage in non-suicidal self-harm. We were able to explore associations with a wide range of prospectively recorded risk factors from different domains. Previous studies have used either cross-sectional study designs (thereby limiting causal inference because they rely on recall of both risk factors and suicidal behaviour) or clinical (or atypical) cohorts with small sample sizes and few risk factor data. Among participants with suicidal thoughts, we found that the strongest predictors of transition to attempts were non-suicidal self-harm, cannabis use, other illicit drug use, exposure to self-harm, and higher levels of the personality type intellect/openness. Among participants with non-suicidal self-harm at baseline, the strongest predictors were cannabis use, other illicit drug use, sleep problems, and lower levels of the personality type extraversion.

**Implications of all the available evidence**

Our findings could help practitioners to identify which adolescents are at greatest risk of attempting suicide in the future, which could lead to improved targeting of prevention and intervention strategies.

An important limitation of previous research is a reliance on cross-sectional studies and the retrospective reporting of both risk factors and suicide-related outcomes. Such studies can be subject to recall bias, and the temporal direction of associations is often unclear. Longitudinal studies adopting an ideation-to-action framework are extremely scarce,^8^, ^22^ and the few existing studies have been done in clinical or atypical samples (university students).^21^, ^23^, ^24^ We aimed to extend previous work by using longitudinal data to explore associations between a comprehensive range of prospectively recorded risk factors and first-time suicide attempts among adolescents with suicidal thoughts and non-suicidal self-harm. Associations were explored in a community-based sample that was more than twice as large as those used in previous longitudinal investigations.

## Methods

### Participants

The Avon Longitudinal Study of Parents And Children (ALSPAC) is an ongoing population-based birth cohort study examining influences on health and development across the life course. The ALSPAC core enrolled sample consists of 14 541 pregnant women resident in the former county of Avon in southwest England (UK), with expected delivery dates between April 1, 1991, and Dec 31, 1992.^25^, ^26^ Of the 14 062 livebirths, 13 798 were singletons or first-born of twins and were alive at 1 year of age. Participants have been followed up regularly since recruitment through questionnaires and research clinics. The study website contains details of all the data that are available through a fully searchable data dictionary. Ethical approval for the study was obtained from the ALSPAC Ethics and Law Committee and the Local Research Ethics Committees.

This investigation is based on the subsample of participants who completed a detailed self-report questionnaire on suicidal thoughts and self-harm at 16 and 21 years of age. Two samples were used for analysis. The first sample included adolescents who reported suicidal thoughts at baseline (n=456), assessed with the question, “Have you ever thought of killing yourself, even if you would not really do it?”. The second sample included adolescents who reported non-suicidal self-harm at baseline (n=569), assessed with the question, “Have you ever hurt yourself on purpose in any way (eg, by taking an overdose of pills, or by cutting yourself)?”. Participants who reported having attempted suicide at the age of 16 years (n=325) were excluded to focus on predictors of first-time suicide attempts.

### Measures

Participants were classified according to whether they reported having ever attempted suicide at 21 years of age. Individuals who indicated having self-harmed, which was assessed by answering “yes” to the question “have you ever hurt yourself on purpose in any way (eg, by taking an overdose of pills or by cutting yourself)?”, were then asked a series of follow-up questions to establish suicidal intent. Participants were classified as having self-harmed with suicidal intent if they either gave the answer “I wanted to die” when asked to give reasons for self-harming or answered “yes” to: “On any of the occasions when you have hurt yourself on purpose, have you ever seriously wanted to kill yourself?”. Suicide attempts were assessed in the same way at 16 years of age.

A description of the risk factors examined in this study is provided in table 1. These risk factors are all known to be associated with self-harm, and their selection was informed by psychological models of suicide and by previous scientific literature. The risk factors included sex, intelligence quotient, executive function, impulsivity, sensation seeking, personality traits, exposure to self-harm in others, life events, early adversity, body dissatisfaction, sleep problems, psychiatric disorders, hopelessness, symptoms of depression, substance use, suicidal plans, and non-suicidal self-harm characteristics. All risk factors were assessed at or before the assessment at 16 years of age.

**Table 1:** Risk factors

		**Age at assessment**	**Measure used**	**Rater**	**Additional information**
**Demographic variables**					
Sex		Birth	Questionnaire item	Mother	None
**Psychosocial variables**					
Intelligence quotient		8 years	Wechsler Intelligence Test for Children, third edition	Child	None
Executive function					
	Updating	8 years	Wechsler Intelligence Test for Children, third edition	Child	Digit span task
	Attentional switching	8 years	The adapted Test-of-Everyday-Attention-for-Children	Child	The dual-attention task of the Sky-Search subtest
	Attentional control	8 years	The adapted Test-of-Everyday-Attention-for-Children	Child	The inhibition aspect of the Opposite Worlds task
Impulsivity		10 years	Stop-signal task	Child	Number of correct trials (lower scores indicate higher impulsivity)
Sensation seeking		16 years	Arnett inventory of sensation-seeking scale	Child	Novelty and intensity subscales
Big five personality dimensions		14 years	International personality item pool	Child	Five subscales (extraversion, agreeableness, conscientiousness, emotional stability, and intellect/openness)
Family self-harm					
	Parent suicide attempt	Repeated eight times from birth to 11 years	Questionnaire item	Mother	Lifetime rating
	Self-harm in family member	16 years	Questionnaire item	Child	Lifetime rating
Friend self-harm		16 years	Questionnaire item	Child	Lifetime rating
Number of life events		16 years	Life events questionnaire	Child	Since age of 12 years
Early adversity*					
	Childhood sexual abuse	Repeated seven times from birth to eight years	Questionnaire item	Mother	None
	Cruelty to children in household	Repeated eight times from birth to 11 years	Questionnaire item	Mother	None
	Being bullied	12 years	Modified version of the bullying and friendship interview schedule	Child	Overt or relational bullying at least once a week over the previous 6 months
Body dissatisfaction		13 years	Questionnaire item		Unhappy or happy over the past year
Sleep problems					
	Waking in the night	15 years	Questionnaire item	Child	Usually wakes at least once a night
	Insufficient sleep	15 years	Questionnaire item	Child	Feels as though usually has too little sleep
**Psychiatric or mental health variables**					
Psychiatric disorder		15 years	DAWBA	Child	None
Depression symptoms		16 years	Short Mood and Feelings Questionnaire	Child	None
Hopelessness		16 years	Community Assessment of Psychic Experience	Child	Two items used: “Have you felt pessimistic about everything?” and “Have you felt as if there is no future for you?”
Substance use					
	Alcohol	15 years	Questionnaire items	Child	Consuming at least four drinks on a typical occasion in the previous 6 months
	Cannabis	15 years	Questionnaire items	Child	At least occasional use
	Smoking	15 years	Questionnaire items	Child	Regular smoking (at least weekly)
	Illicit drugs (other than cannabis)	15 years	Questionnaire items	Child	Past year
Suicidal plans		16 years	Questionnaire item	Child	Lifetime history
Non-suicidal self-harm		16 years	Questionnaire item	Child	Lifetime history
Features of non-suicidal self-harm					
	Frequency	16 years	Questionnaire item	Child	Frequency of self-harm over the past year, coded as no history of self-harm, not in past year, 1–5 times, and ≥6 times. Because of small numbers, we combined together the original categories “once” and “2–5” and the categories “6–10” and “10+”.
	Method of self-harm	16 years	Questionnaire item	Child	Method used on most recent episode. Coded as no history of self-harm, cutting, other, and more than one method.

DAWBA=Development and Well-Being Assessment.

* A composite variable was created because of the low prevalence of individual adversities. This binary (yes or no) measure was derived from responses to questions on sexual abuse, parental cruelty to children in the household, and being bullied.

Additional analyses controlled for the possible confounding effects of child sex and socioeconomic position. Socioeconomic position was assessed by a maternal questionnaire and included average weekly household disposable income recorded at the ages of 3 and 4 years; highest maternal or paternal social class, assessed during pregnancy (professional or managerial, or other); and highest maternal educational attainment, assessed during pregnancy (less than O level, O level, A level, or university degree).

### Statistical analysis

We used logistic regression analyses to examine associations between prospectively recorded risk factors and suicide attempts reported at the age of 21 years. We adjusted for potentially confounding effects of sex and socioeconomic position, but we did not adjust for additional confounders because our aim was to identify potential risk factors for the transition to suicide attempts, rather than to build the most parsimonious prediction model. Continuous risk factors were standardised before analysis to create Z scores with a mean of 0 and an SD of 1.

Our analyses were done on an imputed dataset based on participants who reported suicidal thoughts (n=456) and non-suicidal self-harm (n=569) at baseline. We used multiple imputation by chained equations^27^, ^28^ to generate 50 imputed datasets for each exposure of interest. This method assumes that data are missing at random, whereby any systematic differences between the missing and the observed values can be explained by differences in observed data. Comparison of the estimates from the complete case and imputed data analysis are presented in the appendix. For the non-suicidal group, we did a sensitivity analysis excluding individuals who reported having self-poisoned on the most recent self-harm occasion (appendix). We did all analyses using Stata, version 15.

### Role of the funding source

The funders of the study had no role in study design, data collection, data analysis, data interpretation, or writing of the report. The corresponding author had full access to all the data in the study and had final responsibility for the decision to submit for publication.

## Results

Complete outcome data at 21 years of age were available for 310 participants with suicidal thoughts and 380 participants who had engaged in non-suicidal self-harm (figure). However, by use of the wealth of auxiliary data available in ALSPAC, we were able to impute up to the sample of adolescents with complete data on suicidal thoughts or non-suicidal self-harm at baseline. Participants with and without missing outcome data were found to be similar across a range of demographic variables (appendix); however, several differences were found between responders and non-responders to the self-harm questionnaire completed at 16 years of age (appendix). Participants who responded were more likely to be female and from more highly educated, affluent backgrounds. Findings were broadly consistent in the imputed and complete case analysis. 38 (12%) of 310 participants with suicidal thoughts and 46 (12%) of 380 participants who had engaged in non-suicidal self-harm reported having attempted suicide for the first time by the follow-up at 21 years of age. 107 participants reported both suicidal thoughts and non-suicidal self-harm at 16 years of age. Of these, 22 (21%) reported having attempted suicide by the follow-up at 21 years of age, compared with 32 (1%) of 2283 participants in the subsample who did not report either suicidal thoughts or non-suicidal self-harm at baseline (see the appendix for the prevalence of risk factors in this subgroup). Demographic information for the samples is shown in table 2.

**Figure:**
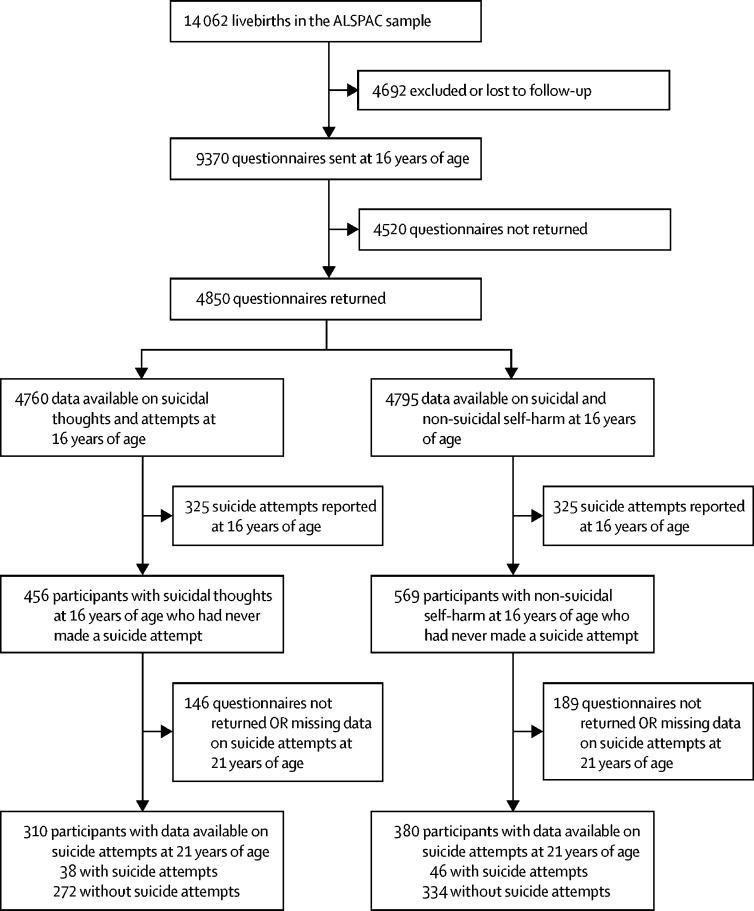
Flow-chart of attrition and self-harm outcomes in the Avon Longitudinal Study of Parents and Children (ALSPAC) birth cohort

**Table 2:** Descriptive information for the complete case sample

	**Sample with suicidal thoughts at baseline**		**Sample with non-suicidal self-harm at baseline**	
	No transition to attempts (n=272)	Transition to attempts (n=38)	No transition to attempts (n=334)	Transition to attempts (n=46)
**Child sex**				
Male	70 (26%)	10 (26%)	59 (18%)	10 (22%)
Female	202 (74%)	28 (74%)	275 (82%)	36 (70%)
**Maternal education**				
A level or degree	142 (52%)	16 (42%)	161 (49%)	21 (46%)
O level	85 (31%)	15 (40%)	117 (36%)	17 (37%)
<O level	44 (16%)	7 (18%)	51 (16%)	8 (17%)
**Parental social class**				
Professional or managerial	174 (66%)	23 (66%)	213 (67%)	26 (59%)
Other	88 (34%)	12 (34%)	106 (33%)	18 (41%)

Data are n (%). Numbers vary because of missing data.

Table 3 shows associations between each risk factor and future suicide attempts among the subsample with suicidal thoughts at baseline. In both unadjusted and adjusted analyses, the strongest evidence for an association was found for cannabis use (adjusted odds ratio [OR] 2·61, 95% CI 1·11–6·14; p=0·029), other illicit drug use (2·47, 1·02–5·96; p=0·045), non-suicidal self-harm (2·78, 1·35–5·74; p=0·0059), and higher levels of the personality type intellect/openness (1·62, 1·06–2·46; p=0·025). There was also weak evidence of an association with exposure to self-harm in others (family member self-harm adjusted OR 2·03, 95% CI 0·93–4·44, p=0·076; friend self-harm 1·85, 0·93–3·69, p=0·081).

**Table 3:** Predictors of incident suicide attempts among adolescents with suicidal thoughts at baseline

		**Total sample**	**No transition to attempts**	**Transition to attempts**	**Unadjusted odds ratio (95% CI)**	**p value**	Adjusted odds ratio*(95% CI)	**p value**
**Sex**								
Male		27%	27%	26%	1 (ref)	..	1 (ref)	..
Female		73%	73%	74%	1·09 (0·51–2·31)	0·822	1·01 (0·47–2·18)	0·975
**Psychosocial variables**								
Intelligence quotient		108·1 (16·4)	108·7 (16·09)	105·8 (16·96)	0·84 (0·58–1·21)	0·347	0·92 (0·60–1·42)	0·716
Executive function								
	Updating	13·2 (2·81)	13·2 (2·81)	12·9 (2·56)	0·89 (0·62–1·26)	0·504	0·94 (0·64–1·37)	0·742
	Attentional switching	11·3 (14·48)	11·5 (15·19)	12·2 (15·7)	1·03 (0·72–1·47)	0·880	0·99 (0·68–1·45)	0·968
	Attentional control	17·9 (15·61)	17·1 (4·33)	17·7 (3·78)	1·13 (0·84–1·52)	0·418	1·11 (0·81–1·51)	0·528
Impulsivity		13·9 (2·38)	14·0 (2·23)	13·4 (3·03)	0·80 (0·57–1·14)	0·221	0·80 (0·56–1·15)	0·228
Sensation seeking								
	Arnett intensity subscale	26·0 (4·76)	25·9 (4·73)	26·5 (4·90)	1·15 (0·83–1·60)	0·412	1·20 (0·85–1·70)	0·290
	Arnett novelty subscale	26·1 (4·50)	26·0 (4·51)	26·8 (4·34)	1·21 (0·86–1·73)	0·275	1·27 (0·88–1·82)	0·201
Big five personality dimensions								
	Extraversion	33·2 (7·81)	33·2 (7·93)	33·2 (7·18)	1·00 (0·69–1·45)	0·990	1·00 (0·68–1·47)	0·992
	Agreeableness	39·3 (4·91)	39·3 (4·92)	39·5 (4·87)	1·05 (0·74–1·47)	0·795	1·08 (0·73–1·59)	0·693
	Conscientiousness	31·2 (5·93)	31·3 (5·88)	30·8 (6·19)	0·93 (0·62–1·40)	0·717	0·90 (0·59–1·38)	0·639
	Emotional stability	28·0 (6·44)	28·1 (6·44)	27·5 (6·40)	0·90 (0·61–1·32)	0·588	0·91 (0·60–1·38)	0·657
	Intellect/openness	36·8 (5·62)	36·5 (5·58)	38·5 (5·52)	1·47 (0·98–2·19)	0·061	1·62 (1·06–2·46)	0·025
Family self-harm		19%	17%	29%	2·04 (0·94–4·41)	0·070	2·03 (0·93–4·44)	0·076
Friend self-harm		61%	59%	72%	1·82 (0·93–3·55)	0·079	1·85 (0·93–3·69)	0·081
Life events		3·9 (2·28)	3·9 (2·31)	3·8 (2·07)	0·92 (0·67–1·28)	0·629	0·90 (0·65–1·24)	0·511
Early adversity		44%	45%	38%	0·73 (0·30–1·74)	0·473	0·75 (0·31–1·81)	0·513
Body dissatisfaction		44%	44%	43%	0·95 (0·47–1·91)	0·874	0·95 (0·45–1·98)	0·883
Sleep problems								
	Waking in the night	55%	55%	58%	1·14 (0·56–2·31)	0·710	1·05 (0·51–2·18)	0·895
	Insufficient sleep	50%	52%	40%	0·62 (0·27–1·41)	0·250	0·59 (0·25–1·39)	0·224
**Psychiatric or mental health variables**								
Any DAWBA diagnosis		12%	11%	14%	1·26 (0·41–3·81)	0·685	1·12 (0·35–3·63)	0·844
Hopelessness		42%	39%	56%	1·61 (0·85–3·07)	0·144	1·65 (0·85–3·18)	0·137
Depression symptoms		11·1 (6·25)	11·1 (6·19)	11·5 (6·62)	1·07 (0·76–1·51)	0·702	1·06 (0·74–1·51)	0·754
Substance use								
	Alcohol, heavy drinking	25%	25%	28%	1·16 (0·46–2·92)	0·749	1·11 (0·43–2·83)	0·830
	Cannabis, at least occasional use	18%	16%	32%	2·46 (1·08–5·62)	0·033	2·61 (1·11–6·14)	0·029
	Smoking, at least weekly	17%	16%	26%	1·78 (0·63–5·00)	0·271	1·70 (0·58–4·97)	0·333
	Other illicit drug use, past year	18%	16%	33%	2·50 (1·06–5·92)	0·037	2·47 (1·02–5·96)	0·045
Suicidal plans		12%	12%	14%	1·17 (0·41–3·37)	0·771	1·15 (0·39–3·41)	0·800
Non-suicidal self-harm		35%	32%	54%	2·47 (1·24–4·90)	0·010	2·78 (1·35–5·74)	0·006
Frequency (percentage of those with non-suicidal self-harm)								
	Not in the last year	42%	39%	51%	1 (ref)	0·401	1 (ref)	0·517
	1–5 times	36%	39%	26%	0·49 (0·17–1·39)	..	0·53 (0·18–1·58)	..
	≥6 times	22%	22%	23%	0·81 (0·27–2·49)	..	0·92 (0·26–3·26)	..
Method used during most recent self-harm episode (percentage of those with non-suicidal self-harm)								
	Cutting	64%	59%	82%	1 (ref)	0·129	1 (ref)	0·107
	Other	14%	Not shown†	Not shown†	0·57 (0·16–2·08)	..	0·44 (0·11–1·83)	..
	Multiple methods	23%	Not shown†	Not shown†	0·16 (0·02–1·18)	..	0·15 (0·02–1·16)	..

Data are percentages or mean (SD). Sample numbers not shown because percentages are based on imputed data (n=456). DAWBA=Development and Well-Being Assessment.

* Adjusted for sex and socioeconomic position.

† Data censored to prevent disclosure due to small cell counts; continuous variables were standardised before analysis.

Table 4 shows associations between each risk factor and future suicide attempts among the subsample with non-suicidal self-harm at baseline. In both unadjusted and adjusted analyses, the strongest evidence predicting the transition to suicide attempts was found for cannabis use (adjusted OR 2·14, 95% CI 1·04–4·41; p=0·038), other illicit drug use (2·17, 1·10–4·27; p=0·025), and insufficient sleep (1·97, 1·02–3·81; p=0·043). There was also weak evidence of an association with waking in the night (adjusted OR 1·91, 95% CI 0·93–4·44; p=0·069) and lower levels of the personality type extraversion (0·71, 0·49–1·03; p=0·068).

**Table 4:** Predictors of incident suicide attempts among adolescents with non-suicidal self-harm at baseline

		**Total sample**	**No transition to attempts**	**Transition to attempts**	**Unadjusted odd ratio (95% CI)**	**p value**	Adjusted odds ratio*(95% CI)	**p value**
**Sex**								
Male		20%	20%	22%	1 (ref)	..	1 (ref)	..
Female		80%	80%	78%	0·87 (0·42–1·80)	0·711	0·82 (0·39–1·72)	0·596
**Psychosocial variables**								
Total intelligence quotient (age 8 years)		109·3 (14·79)	109·4 (14·66)	108·4 (15·47)	0·94 (0·67–1·32)	0·711	0·99 (0·67–1·46)	0·944
Executive function								
	Updating	13·4 (2·74)	13·5 (2·77)	13·2 (2·52)	0·88 (0·64–1·20)	0·421	0·90 (0·64–1·27)	0·552
	Attentional switching	10·8 (11·74)	10·7 (11·82)	11·1 (11·23)	0·99 (0·67–1·46)	0·959	0·98 (0·65–1·48)	0·931
	Attentional control	16·9 (3·71)	16·9 (3·67)	17·1 (3·89)	1·06 (0·77–1·45)	0·725	1·02 (0·74–1·42)	0·886
Impulsivity		13·7 (2·59)	13·8 (2·54)	13·5 (2·81)	0·93 (0·64–1·33)	0·672	0·92 (0·64–1·34)	0·670
Sensation seeking								
	Arnett intensity subscale	26·2 (4·81)	26·2 (4·78)	26·2 (4·97)	1·01 (0·77–1·34)	0·938	0·97 (0·72–1·31)	0·848
	Arnett novelty subscale	26·6 (4·35)	26·6 (4·30)	26·9 (4·66)	1·07 (0·81–1·41)	0·625	1·06 (0·79–1·40)	0·711
Big five personality dimensions								
	Extraversion	35·3 (6·99)	35·6 (6·83)	33·4 (7·53)	0·73 (0·52–1·03)	0·072	0·71 (0·49–1·03)	0·068
	Agreeableness	39·1 (4·89)	39·1 (4·90)	39·1 (4·82)	0·99 (0·72–1·37)	0·965	1·04 (0·73–1·47)	0·837
	Conscientiousness	30·4 (6·11)	30·5 (6·18)	30·3 (5·70)	0·97 (0·71–1·32)	0·826	0·95 (0·70–1·31)	0·773
	Emotional stability	28·9 (6·52)	29·1 (6·49)	27·7 (6·52)	0·80 (0·55–1·17)	0·254	0·79 (0·53–1·17)	0·237
	Intellect/openness	36·9 (5·83)	36·7 (5·83)	37·9 (5·73)	1·24 (0·88–1·75)	0·222	1·25 (0·87–1·81)	0·221
Family self-harm		18%	18%	23%	1·38 (0·66–2·89)	0·390	1·42 (0·66–3·05)	0·364
Friend self-harm		76%	76%	75%	0·94 (0·49–1·79)	0·851	0·97 (0·50–1·89)	0·936
Life events		3·7 (2·28)	3·7 (2·30)	3·8 (2·13)	1·07 (0·79–1·45)	0·680	1·09 (0·80–1·49)	0·573
Early adversity		35%	33%	36%	1·13 (0·56 2·30)	0·727	1·14 (0·56–2·35)	0·712
Body dissatisfaction		48%	49%	38%	0·63 (0·33–1·20)	0·160	0·63 (0·33–1·23)	0·176
Sleep problems								
	Waking in the night	55%	52%	67%	1·94 (1·01–3·73)	0·047	1·91 (0·95–3·84)	0·069
	Insufficient sleep	41%	39%	55%	1·90 (1·00–3·59)	0·049	1·97 (1·02–3·81)	0·043
**Psychiatric or mental health variables**								
Any DAWBA diagnosis		9%	Not shown†	Not shown†	0·56 (0·13–2·35)	0·428	0·54 (0·13–2·30)	0·403
Hopelessness		29%	28%	32%	1·15 (0·58–2·26)	0·691	1·20 (0·60–2·40)	0·603
Depressive symptoms		8·7 (5·94)	8·7 (5·89)	8·8 (6·27)	1·01 (0·73–1·38)	0·975	1·00 (0·73–1·38)	0·996
Substance use								
	Alcohol, heavy drinking	29%	32%	27%	0·79 (0·40–1·58)	0·510	0·77 (0·38–1·54)	0·452
	Cannabis, at least occasional use	20%	18%	32%	2·15 (1·07–4·32)	0·032	2·14 (1·04–4·41)	0·038
	Smoking, at least weekly	16%	14%	26%	2·09 (0·86–5·05)	0·101	2·07 (0·83–5·13)	0·116
	Other illicit drug use, past year	26%	24%	40%	2·15 (1·10–4·19)	0·025	2·17 (1·10–4·27)	0·025
Suicidal plans		4%	4%	8%	2·17 (0·63–7·48)	0·218	2·25 (0·62–8·12)	0·215
Non-suicidal self-harm frequency								
	Not in the last year	48%	48%	51%	1 (ref)	0·622	1 (ref)	0·599
	1–5 times	39%	40%	34%	0·80 (0·42–1·51)	..	0·83 (0·44–1·58)	..
	≥6 times	13%	13%	16%	1·19 (0·53–2·65)	..	1·29 (0·57–2·92)	..
Non-suicidal self-harm method used during most recent self-harm episode								
	Cutting	72%	71%	79%	1 (ref)	0·529	1 (ref)	0·554
	Other	16%	16%	13%	0·70 (0·29–1·69)	..	0·67 (0·27–1·65)	..
	Multiple methods	12%	13%	9%	0·62 (0·21–1·83)	..	0·66 (0·22–1·99)	..

Data are percentages or mean (SD). Sample numbers not shown because percentages are based on imputed data (n=569). DAWBA=Development and Well-Being Assessment.

* Adjusted for sex and socioeconomic position.

† Data censored to prevent disclosure because of small cell counts; continuous variables were standardised before analysis.

A small proportion (15 [4%] of 380) of adolescents in the non-suicidal self-harm group reported having self-poisoned on the most recent self-harm occasion; however, sensitivity analysis excluding these individuals did not change the pattern of results (appendix).

## Discussion

To our knowledge, this is the largest longitudinal study to explore the transition to suicide attempts among adolescents with suicidal thoughts or non-suicidal self-harm. We identified several risk factors that predicted future suicide attempts in these high-risk groups. Among participants with suicidal thoughts at 16 years of age, future risk of suicide attempt was associated with non-suicidal self-harm history, cannabis use, other illicit drug use, higher intellect/openness score, and exposure to self-harm in others. This finding is consistent with a cross-sectional analysis of this cohort,^9^ which found substance use and exposure to self-harm differentiated between adolescents with suicidal thoughts and those who had attempted suicide at age 16 years. Both cannabis and other illicit drug use also predicted the transition to attempts among participants with non-suicidal self-harm at baseline, along with a lower extraversion score and sleep difficulties.

Although some differences were found in the predictors of transition for participants with suicidal thoughts and those with non-suicidal self-harm at baseline, other illicit drug use and cannabis use were identified in both samples, suggesting that these factors might be particularly robust predictors of future suicide attempt risk. Consistent with our findings, a previous meta-analysis^5^ found drug use moderately distinguished between adolescents with suicidal thoughts and attempts. However, a separate meta-analysis^20^ did not find an association with attempts among adolescents with non-suicidal self-harm. It is possible that substances such as cannabis and other illicit drugs increase suicide capability by lowering inhibitions and impairing decision making. It is also possible that drug use leads to mental illness over time, and this mental illness leads to suicide attempts. Alternatively, substance use might be a proxy for particular types of coping in response to stress that are maladaptive. There is also evidence to suggest that there might be a bidirectional relationship; several longitudinal studies^29^, ^30^, ^31^, ^32^, ^33^ have reported an association between adolescent self-harm and substance use problems in adulthood. Notably, we did not find evidence for an association with alcohol use or smoking in either sample, which highlights the importance of exploring relationships with different substances independently. Future research should explore whether associations differ for different forms of illicit drug use (eg, injection drug use).

Previous research suggests that non-suicidal self-harm is a robust predictor of future suicide attempts;^15^, ^16^, ^17^, ^18^, ^19^ however, non-suicidal self-harm has rarely been considered within an ideation-to-action framework. Our study extends previous work by demonstrating that non-suicidal self-harm is specifically associated with the transition from suicidal thinking to action. Several explanations for this association are possible, including shared neurobiological vulnerability to self-harm, an increased risk of social exclusion or mental illness as a result of non-suicidal self-harm,^34^ or a direct effect on reducing the inhibition to attempt suicide in the face of suicidal thoughts.^2^ Our findings are in line with those of a previous prospective community study of adolescents^35^ and indicate that those individuals who report both suicidal thoughts and non-suicidal self-harm might be an especially high-risk group. We found that approximately 1 in 5 (21%) of the adolescents who reported both suicidal thoughts and non-suicidal self-harm at baseline went on to make a suicide attempt, which compares with only 1% of those who did not report either of these behaviours. Despite the low prevalence, it is notable that this group accounted for approximately a quarter of participants who attempted suicide over the follow-up. In contrast to some previous studies,^20^, ^21^ we did not find characteristics of non-suicidal self-harm (such as method and frequency) to be strong predictors of future suicide attempts. This difference could be due to methodological differences in sample or definition of non-suicidal self-harm: for example, the timeframe of assessment (past year *vs* lifetime) or method choice (lifetime *vs* most recent). Alternatively, we might have been underpowered to detect effects; however, our sample size is more than twice as large as the only other longitudinal study^21^ exploring predictors of concurrent and future suicide attempts among adolescents with non-suicidal self-harm.

Other factors that were associated with future suicide attempts among participants with non-suicidal self-harm included sleep problems and a lower extraversion score. Both of these factors have been associated with suicidal behaviour in previous research;^36^, ^37^, ^38^, ^39^ however, our study is the first to explore prospectively the role of sleep difficulties and personality traits in the transition to suicide attempts over time. It might be that individuals who are less extraverted are more socially disconnected, which has been shown to predict future suicide attempts in a sample of university students with non-suicidal self-harm.^21^ Sleep problems could affect feelings of social connection by impairing an individual's ability and motivation to interact with others.^40^, ^41^ They might also have a more direct effect on suicide risk, leading to increased distress at a time when fewer social supports are available.

A growing number of cross-sectional studies have found that exposure to self-harm in others differentiates between adolescents with suicidal ideation and attempts.^9^, ^10^, ^12^, ^13^, ^42^ In this study, we found weak evidence to suggest that exposure to self-harm also predicts future suicide attempts in adolescents who have thought about suicide, but not among those who have been engaged in non-suicidal self-harm. One explanation is that self-harm exposure might increase the capability of suicide among adolescents with suicidal thoughts by increasing the salience and acceptability of self-harm (eg, increased awareness of self-harm as an option, its functional utility, and knowledge of methods),^43^ whereas those who have engaged in non-suicidal self-harm are already aware of self-harm methods. Further research will need to investigate the mechanisms by which exposure to self-harm in others increases the risk of suicide attempts. Potential candidate mechanisms include genetic influences, social transmission, imitation, and assortative relating among people at high risk.

It might appear surprising that we did not find evidence of an association for several well established suicide risk factors, including depression symptoms, psychiatric disorder, suicidal plans, and impulsivity. However, our results are consistent with previous research^5^, ^20^ that has suggested that these factors appear to be associated with suicide attempts because they are associated with the development of suicidal thoughts or non-suicidal self-harm, but are not involved in the transition. An alternative methodological explanation for this negative finding could be that we (in common with other large epidemiological studies) did not measure symptoms immediately before the suicide attempt, when there might have been a stronger association than at 16 years of age. The CIs for some predictors are also wide, and it is possible there is an association that we were underpowered to detect.

This study has many strengths, including the large population-based sample, longitudinal design, and ability to explore a wide range of prospectively recorded risk factors. The vast majority of research in this area has been cross-sectional, and therefore limited by retrospective reporting of both risk factors and outcomes. We also excluded people with a previous suicide attempt at baseline, which enabled us to establish the direction of effects between our measures and ensure that we were not modelling risk for repeat suicide attempts.

There are also several limitations to consider. First, it cannot be assumed that the associations identified in this study are causal. We adjusted only for two confounding variables (sex and socioeconomic position); however, it was not our aim to identify independent predictors, and to examine this adequately would require a separate theory-driven analytical model for each exposure. This analysis was beyond the scope of the current paper, but is an important area for future research. Second, information was not available on the date of the first suicide attempt. We therefore focused on risk factors that occurred at or before the age 16 years' assessment to ensure that they preceded the outcome. 5 years is a relatively long follow-up period, and risk factors that predict the transition to suicide attempts over the short term might differ from those that predict over the long term. Newly emerging methods of data collection, such as Ecological Momentary Assessment, could be used in future studies to explore predictors of transitions over a shorter timeframe (ie, hours, days, or weeks). Third, we excluded individuals who had attempted suicide before the age of 16 years so that we could examine predictors of incident suicide attempts. Although we consider this approach to be a strength of the study, it is possible that it weakened associations with some of our risk factors. For example, if a particular risk factor is strongly associated with suicide attempts (eg, suicidal plans), then it is more likely that individuals with that risk factor would have already attempted suicide, and therefore been excluded from the analyses. This means that our findings might not be applicable to individuals who have already attempted suicide by the age of 16 years. However, identifying individuals who will make a first attempt in late adolescence or young adulthood is important, because this is the age at which hospital presentations for self-harm are at their highest.^44^ Further longitudinal research is needed to explore whether there are differences in the risk factors for incident and repeat suicide attempts among individuals with current suicidal ideation. Fourth, we did not correct for multiple testing as analyses were exploratory. Our results are therefore in need of replication, given the large number of tests done. Finally, as with all longitudinal studies, there was some attrition over time that might have biased our complete case analyses. However, findings were similar using imputed data, suggesting that the effects of this potential bias were not substantial. Although we cannot say with certainty that our data are missing at random, ALSPAC contains a wealth of auxiliary data, which increases the plausibility of this assumption. There were also some differences between those individuals who did and did not respond to the age 16 years' self-harm questionnaire and this non-random response might limit the generalisability of our results.

Identification of factors that predict the transition from suicidal thoughts or non-suicidal self-harm to suicide attempts is crucial for improved suicide prediction and prevention. Although results of existing cross-sectional research have provided important information about the factors that differentiate between individuals with suicidal thoughts or non-suicidal self-harm and those with attempts, longitudinal studies such as this are required to investigate whether the identified factors predict the transition to attempts over time. Our findings suggest that asking about factors such as substance use, non-suicidal self-harm, sleep, personality traits, and exposure to self-harm might help clinicians to identify which adolescents are at greatest risk of attempting suicide in the future.
