# Supplementary material for: Predictors of future suicide attempt among adolescents with suicidal thoughts or non-suicidal self-harm: a population-based birth cohort study
Source: Lancet Psychiatry. 2019 Apr;6(4):327–37. doi: 10.1016/S2215-0366(19)30030-6 (PMC6494973; doi:10.1016/S2215-0366(19)30030-6)
Supplement: Supplementary appendix [file mmc1.pdf]

THE LANCET
Psychiatry

Supplementary appendix

This appendix formed part of the original submission and has been peer reviewed. We post it as supplied by the authors.

Supplement to: Mars B, Heron J, Klonsky ED, et al. Predictors of future suicide attempt among adolescents with suicidal thoughts or non-suicidal self-harm: a population-based birth cohort study. *Lancet Psychiatry* 2019; published online March 14.
http://dx.doi.org/10.1016/S2215-0366(19)30030-6.

eTable 1: Comparison of those with complete and incomplete data on suicide attempts at 21 years (Comparison amongst those with suicidal thoughts at baseline, *N*=456)

eTable 2: Comparison of those with complete and incomplete data on suicide attempts at 21 years (Comparison amongst those with non-suicidal self-harm at baseline, *N*=569)

eTable 3 Comparison of responders and non-responders to the self-harm questionnaire at 16 years by key demographic variables (Comparison amongst those invited to participate in the age 16 year questionnaire, *N*=9,370)

eTable 4: Predictors of incident suicide attempts amongst those with suicidal thoughts at baseline: Comparison of complete case and imputed data

eTable 5: Predictors of incident suicide attempts amongst those with non-suicidal self-harm at baseline: Comparison of complete case and imputed data

eTable 6: Description of risk factors in the subsample with no suicidal thoughts or attempts at age 16 years

eTable 7: Predictors of incident suicide attempts amongst those with non-suicidal self-harm at baseline: Sensitivity analysis removing those who reported having self-poisoned on the most recent self-harm occasion

eTable 1: Comparison of those with complete and incomplete data on suicide attempts at 21 years (Comparison amongst those with suicidal thoughts at baseline, *N*=456)

Variable	Description	*N*	Complete data on suicide attempts n=310	Incomplete data on suicide attempts n=146	*P* value [a]
Child sex	Male	123	80 (25·8%)	43 (29·5%)	0·413
	Female	333	230 (74·2%)	103 (70·6%)	
Parity	First born	199	131 (43·0%)	68(48·9%)	0·384
	Second born	171	119 (39·0%)	52 (37·4%)	
	Third born plus	74	55 (18·0%)	19 (13·7%)	
Home overcrowding	≤1 person/room	424	293 (97·7%)	131(94·9%)	0·130
	>1 person/room	14	7 (2·3%)	7 (5·1%)	
Maternal education	Degree	94	69 (22·3%)	25 (18·5%)	0·377
	A level	121	89 (28·8%)	32 (23·7%)	
	O level	150	100 (32·4%)	50 (37·0%)	
	< O level	79	51 (16·5%)	28 (20·7%)	
Equivalised household income	1st quintile	104	71 (25·0%)	33 (25·0%)	0·114
	2nd quintile	94	72 (25·4%)	22 (16·7%)	
	3rd quintile	89	62 (21·8%)	27 (20·5%)	
	4th quintile	70	46 (16·2%)	24 (18·2%)	
	5th quintile	59	33 (11·6%)	26 (19·7%)	
Parental social class	Professional/managerial	278	197 (66·3%)	81 (61·8%)	0·369
	other	150	100 (33·7%)	50 (38·2%)	

[a] Chi-Square test of the association between missing data and categorical exposures
Numbers vary due to missing data

eTable 2: Comparison of those with complete and incomplete data on suicide attempts at 21 years (Comparison amongst those with non-suicidal self-harm at baseline, *N*=569)

Variable	Description	*N*	Complete data on suicide attempts n=380	Incomplete data on suicide attempts n=189	*P* value [a]
Child sex	Male	116	69 (18·2%)	47 (24·9%)	0·061
	Female	453	311 (81·8%)	142 (75·1%)	
Parity	First born	265	182 (49·4%)	83(44·9%)	0·579
	Second born	214	139 (37·8%)	75 (40·5%)	
	Third born plus	74	47 (12·8%)	27 (36·5%)	
Home overcrowding	≤1 person/room	533	360 (97·8%)	173(96·1%)	0·248
	>1 person/room	15	8 (2·2%)	7 (3·9%)	
Maternal education	Degree	133	91 (24·3%)	42 (23·3%)	0·898
	A level	131	91 (24·3%)	40 (22·2%)	
	O level	204	134 (35·7%)	70 (38·9%)	
	< O level	87	59 (15·7%)	28 (15·6%)	
Equivalised household income	1st quintile	130	86 (24·8%)	44 (25·4%)	0·449
	2nd quintile	117	85 (24·5%)	32 (18·5%)	
	3rd quintile	113	70 (20·2%)	43 (24·9%)	
	4th quintile	90	62 (17·9%)	28 (16·2%)	
	5th quintile	70	44 (12·7%)	26 (15·0%)	
Parental social class	Professional/managerial	349	239 (65·8%)	110(64·7%)	0·797
	other	184	124 (34·2%)	60 (35·3%)	

[a] Chi-Square test of the association between missing data and categorical exposures
Numbers vary due to missing data

eTable 3: Comparison of responders and non-responders to the self-harm questionnaire at 16 years by key demographic variables (Comparison amongst those invited to participate in the age 16 year questionnaire, *N*=9,370)

**Variable**	**Description**	***N***	**No data on suicidal thoughts and attempts [a]**	**Data on suicidal thoughts and attempts**	***P* value [b]**
Child sex	Male	4,628	2,672 (58·1%)	1,956 (41·0%)	<0·001
	Female	4,742	1,956 (41·9%)	2,816 (59·0%)	
Parity	First born	4,060	1,789 (41·5%)	2,271 (48·8%)	<0·001
	Second born	3,199	1,571 (36·5%)	1,628 (35·0%)	
	Third born plus	1,700	948 (22·0%)	752 (16·2%)	
Home overcrowding	≤1 person/room	8,434	3,994 (93·1%)	4,440 (96·6%)	<0·001
	>1 person/room	453	296 (6·9%)	157 (3·4%)	
Maternal education	Degree	1,306	365 (8·7%)	941 (20·2%)	<0·001
	A level	2,206	906 (21·5%)	1,300 (28·0%)	
	O level	3,106	1,561 (37·0%)	1,545 (33·2%)	
	< O level	2,246	1,383 (32·8%)	863 (18·6%)	
Equivalised household income	1st quintile	1,724	599 (16·7%)	1,125 (25·9%)	<0·001
	2nd quintile	1,657	651 (18·2%)	1,006 (23·1%)	
	3rd quintile	1,612	753 (21·0%)	859 (19·8%)	
	4th quintile	1,519	762 (21·3%)	757 (17·4%)	
	5th quintile	1,417	818 (22·8%)	599 (13·8%)	
Parental social class	Professional/managerial	4,870	1,979 (50·6%)	2,891 (64·9%)	<0·001
	other	3,499	1,933 (49·4%)	1,566 (35·1%)	

[a] Includes those who were sent but did not return the self-harm questionnaire, and those who returned the questionnaire but did not respond to the suicidal thoughts or attempts items

[b] Chi-Square test of the association between missing data and categorical exposures

Numbers vary due to missing data

eTable 4: Predictors of incident suicide attempts amongst those with suicidal thoughts at baseline: Comparison of complete case and imputed data

Exposure	Complete case n=310		Imputed sample n=456	
	**Adjusted for sex and SES OR (95% CI)**	**P value**	**Adjusted for sex and SES OR (95% CI)**	**P value**
Female sex	0·85 (0·37, 1·96)	0·698	1·01 (0·47, 2·18)	0·975
**Psychosocial variables**				
IQ	0·91 (0·58, 1·42)	0·678	0·92 (0·60, 1·42)	0·716
Executive function				
Updating	0·89 (0·57, 1·39)	0·616	0·94 (0·64, 1·37)	0·742
Attentional-switching	1·08 (0·74, 1·58)	0·700	0·99 (0·68, 1·45)	0·968
Attentional-control	1·36 (0·85, 2·18)	0·203	1·11 (0·81, 1·51)	0·528
Impulsivity	0·88 (0·60, 1·31)	0·537	0·80 (0·56, 1·15)	0·228
Sensation-seeking				
Arnett intensity subscale	1·12 (0·76, 1·66)	0·555	1·20 (0·85, 1·70)	0·290
Arnett novelty subscale	1·13 (0·77, 1·66)	0·539	1·27 (0·88, 1·82)	0·201
Big-5 personality dimensions				
Extraversion	0·84 (0·53, 1·32)	0·981	1·00 (0·68, 1·47)	0·992
Agreeableness	1·08 (0·65, 1·78)	0·776	1·08 (0·73, 1·59)	0·693
Conscientiousness	1·01 (0·64, 1·58)	0·981	0·90 (0·59, 1·38)	0·639
Emotional stability	0·96 (0·60, 1·54)	0·874	0·91 (0·60, 1·38)	0·657
Intellect/openness	1·56 (0·95, 2·54)	0·076	1·62 (1·06, 2·46)	0·025
Family self-harm	2·46 (1·02, 5·93)	0·045	2·03 (0·93, 4·44)	0·076
Friend self-harm	1·77 (0·79, 3·98)	0·166	1·85 (0·93, 3·69)	0·081
Life events	0·80 (0·55, 1·19)	0·271	0·90 (0·65, 1·24)	0·511
Early adversity	0·81 (0·32, 2·09)	0·668	0·75 (0·31, 1·81)	0·513
Body dissatisfaction	0·94 (0·41, 2·15)	0·880	0·95 (0·45, 1·98)	0·883
Sleep problems				
Waking in the night	1·14 (0·47, 2·73)	0·776	1·05 (0·51, 2·18)	0·895
Insufficient sleep	0·47 (0·19, 1·12)	0·088	0·59 (0·25, 1·39)	0·224
**Psychiatric/mental health variables**				
Any DAWBA diagnosis	1·07 (0·28, 4·10)	0·927	1·12 (0·35, 3·63)	0·844
Hopelessness	2·20 (0·99, 4·87)	0·052	1·65 (0·85, 3·18)	0·137
Depressive symptoms	1·11 (0·74, 1·65)	0·612	1·06 (0·74, 1·51)	0·754
Substance use				
Alcohol, heavy drinking	1·37 (0·49, 3·81)	0·543	1·11 (0·43, 2·83)	0·830
Cannabis, at least occasional use	2·99 (1·01, 8·81)	0·047	2·61 (1·11, 6·14)	0·029
Smoking, at least weekly	1·85 (0·54, 6·42)	0·330	1·70 (0·58, 4·97)	0·333
Illicit drug use (post year)	3·45 (1·29, 9·24)	0·014	2·47 (1·02, 5·96)	0·045
Suicidal plans	1·58 (0·49, 5·11)	0·448	1·15 (0·39, 3·41)	0·800
NSSH	3·73 (1·71, 8·12)	0·001	2·78 (1·35, 5·74)	0·006
NSSH frequency				
Not in the last year	(reference)	0·561	(reference)	0·517
1-5 times	0·59 (0·16, 2·14)		0·53 (0·18, 1·58)	
6+ times	1·26 (0·32, 5·00)		0·92 (0·26, 3·26)	
NSSH method used during most recent self-harm episode				
Cutting	(reference)	0·049	(reference)	0·107
Other	0·29 (0·05, 1·59)		0·44 (0·11, 1·83)	
Multiple methods	0·14 (0·02, 1·25)		0·15 (0·02, 1·16)	

NSSH: non-suicidal self-harm

The number of individuals with missing data was 0 for gender; 58 for IQ; 60 for updating; 69 for attentional switching; 52 for attentional control; 66 for impulsivity; 5 for sensation seeking; 74 for extraversion; 77 for agreeableness; 81 for conscientiousness; 84 for emotional stability; 78 for intellect/openness; 37 for family self-harm; 0 for friend self-harm; 0 for life events; 115 for adversity; 74 for body dissatisfaction; 110 for waking in

the night; 90 for insufficient sleep; 74 for psychiatric disorder; 15 for hopelessness; 4 for depressive symptoms; 81 for alcohol; 77 for cannabis; 106 for smoking; 84 for illicit drug use; 29 for suicidal plans; 0 for NSSH

eTable 5: Predictors of incident suicide attempts amongst those with non-suicidal self-harm at baseline: Comparison of complete case and imputed data

Exposure	Complete case n=380		Imputed sample n=569	
	Adjusted for sex and SES OR (95% CI)	P value	Adjusted for sex and SES OR (95% CI)	P value
Female sex	0·68 (0·30, 1·54)	0·354	0·82 (0·39, 1·72)	0·596
**Psychosocial variables**				
IQ	1·03 (0·69, 1·53)	0·892	0·99 (0·67, 1·46)	0·944
Executive function				
Updating	0·90 (0·61, 1·32)	0·583	0·90 (0·64, 1·27)	0·552
Attentional-switching	0·96 (0·66, 1·40)	0·836	0·98 (0·65, 1·48)	0·931
Attentional-control	0·94 (0·66, 1·33)	0·715	1·02 (0·74, 1·42)	0·886
Impulsivity	1·04 (0·71, 1·52)	0·836	0·92 (0·64, 1·34)	0·670
Sensation-seeking				
Arnett intensity subscale	0·92 (0·65, 1·31)	0·652	0·97 (0·72, 1·31)	0·848
Arnett novelty subscale	0·97 (0·70, 1·36)	0·862	1·06 (0·79, 1·40)	0·711
Big-5 personality dimensions				
Extraversion	0·58 (0·39, 0·87)	0·008	0·71 (0·49, 1·03)	0·068
Agreeableness	1·01 (0·69, 1·49)	0·943	1·04 (0·73, 1·47)	0·837
Conscientiousness	1·01 (0·69, 1·48)	0·970	0·95 (0·70, 1·31)	0·773
Emotional stability	0·84 (0·57, 1·25)	0·397	0·79 (0·53, 1·17)	0·237
Intellect/openness	1·12 (0·77, 1·63)	0·545	1·25 (0·87, 1·81)	0·221
Family self-harm	1·68 (0·75, 3·76)	0·206	1·42 (0·66, 3·05)	0·364
Friend self-harm	0·96 (0·45, 2·06)	0·924	0·97 (0·50, 1·89)	0·936
Life events	0·98 (0·70, 1·36)	0·887	1·09 (0·80, 1·49)	0·573
Early adversity	0·92 (0·38, 2·21)	0·853	1·14 (0·56, 2·35)	0·712
Body dissatisfaction	0·66 (0·32, 1·36)	0·257	0·63 (0·33, 1·23)	0·176
Sleep problems				
Waking in the night	2·06 (0·92, 4·58)	0·077	1·91 (0·95, 3·84)	0·069
Insufficient sleep	1·87 (0·90, 3·89)	0·094	1·97 (1·02, 3·81)	0·043
**Psychiatric/mental health variables**				
Any DAWBA diagnosis	0·62 (0·13, 2·89)	0·547	0·54 (0·13, 2·30)	0·403
Hopelessness	1·27 (0·61, 2·66)	0·525	1·20 (0·60, 2·40)	0·603
Depressive symptoms	0·98 (0·71, 1·37)	0·923	1·00 (0·73, 1·38)	0·996
Substance use				
Alcohol, heavy drinking	0·80 (0·35, 1·82)	0·591	0·77 (0·38, 1·54)	0·452
Cannabis, at least occasional use	2·30 (1·01, 5·23)	0·047	2·14 (1·04, 4·41)	0·038
Smoking, at least weekly	1·98 (0·73, 5·38)	0·178	2·07 (0·83, 5·13)	0·116
illicit drug use (post year)	2·39 (1·11, 5·18)	0·026	2·17 (1·10, 4·27)	0·025
Suicidal plans	2·18 (0·56, 8·56)	0·263	2·25 (0·62, 8·12)	0·215
NSSH frequency				
Not in the last year	(reference)	0·711	(reference)	0·599
1-5 times	1·03 (0·49, 2·16)		0·83 (0·44, 1·58)	
6+ times	1·46 (0·59, 3·64)		1·29 (0·57, 2·92)	
NSSH method used during most recent self-harm episode				
Cutting	(reference)	0·191	(reference)	0·554
Other	0·47 (0·16, 1·42)		0·67 (0·27, 1·65)	
More than one	0·42 (0·10, 1·85)		0·66 (0·22, 1·99)	

NSSH: non-suicidal self-harm
The number of individuals with missing data was 0 for gender; 56 for IQ; 59 for updating; 75 for attentional switching; 64 for attentional control; 72 for impulsivity; 4 for intensity seeking; 9 for novelty seeking; 82 for extraversion; 83 for agreeableness; 84 for conscientiousness; 89 for emotional stability; 87 for intellect/openness; 37 for family self-harm; 3 for friend self-harm; 0 for life events; 136 for adversity; 50 for body dissatisfaction; 113 for waking in the night; 103 for insufficient sleep; 74 for psychiatric disorder; 17 for hopelessness; 2 for depressive symptoms; 80 for alcohol; 75 for cannabis; 121 for smoking; 86 for illicit drug use; 2 for suicidal plans; 5 for NSSH frequency; 4 for NSSH method·

eTable 6: Description of risk factors in the subsample with no suicidal thoughts or attempts at age 16 years: complete case sample

**Exposure (age of assessment)**	**Number (%) or Mean (SD)**<br><br>**n=2283**
Female sex, (%)	57·16%
**Psychosocial variables**	
Total IQ (age 8, mean (SD)	109·3 (15·86)
Executive function, mean (SD)	
Updating	13·4 (2·94)
Attentional-switching	11·1 (15·10)
Attentional-control	16·9 (5·28)
Impulsivity, mean (SD)	13·7 (2·54)
Sensation-seeking, mean (SD)	
Arnett intensity subscale	25·5 (4·43)
Arnett novelty subscale	25·7 (4·28)
Big-5 personality dimensions, mean (SD)	
Extraversion	34·8 (6·97)
Agreeableness	38·5 (4·97)
Conscientiousness	33·1 (5·77)
Emotional stability	33·0 (6·27)
Intellect/openness	36·4 (5·47)
Family self-harm, (%)	5·76%
Friend self-harm, (%)	30·5%
Life events, mean (SD)	2·6 (1·89)
Early adversity, (%)	24·4%
Body dissatisfaction, *(%)*	28·5%
Sleep problems, (%)	
Waking in the night	38·5%
Insufficient sleep	31·8%
**Psychiatric/mental health variables**	
Any DAWBA diagnosis, (%)	2·6%
Hopelessness, (%)	9·2%
Depressive symptoms, mean (SD)	4·3 (4·13)
Substance use, (%)	
Alcohol, heavy drinking	15·2%
Cannabis, at least occasional use	5·3%
Smoking, at least weekly	5·1%
illicit drug use (post year)	7·5%
Suicidal plans, (%)	0%

Sample includes 2283 with data on suicidal thoughts and non-suicidal self-harm at 16 years, and suicidal attempts at age 21 years

The number of individuals with missing data was 0 for gender; 372 for IQ; 411 for updating; 438 for attentional switching; 416 for attentional control; 447 for impulsivity; 58 for intensity seeking; 55 for novelty seeking; 483 for extraversion; 521 for agreeableness; 573 for conscientiousness; 532 for emotional stability; 515 for intellect/openness; 199 for family self-harm; 10 for friend self-harm; 2 for life events; 852 for adversity; 292 for body dissatisfaction; 670 for waking in the night; 606 for insufficient sleep; 481 for psychiatric disorder; 81 for hopelessness; 43 for depressive symptoms; 547 for alcohol; 502 for cannabis; 746 for smoking; 580 for illicit drug use; 0 for suicidal plans

eTable 7: Predictors of incident suicide attempts amongst those with non-suicidal self-harm at baseline: Sensitivity analysis removing those who reported having self-poisoned on the most recent self-harm occasion

**Exposure**	**Complete case n=380**	
	**Adjusted for sex and SES OR (95% CI)**	**P value**
Female sex	0·67 (0·29, 1·53)	0·338
**Psychosocial variables**		
IQ	1·07 (0·71, 1·62)	0·736
Executive function		
Updating	0·95 (0·65, 1·38)	0·779
Attentional-switching	0·93 (0·62, 1·41)	0·746
Attentional-control	0·96 (0·67, 1·37)	0·816
Impulsivity	1·00 (0·69, 1·46)	0·995
Sensation-seeking		
Arnett intensity subscale	0·91 (0·64, 1·29)	0·593
Arnett novelty subscale	1.02 (0·72, 1·43)	0·919
Big-5 personality dimensions		
Extraversion	0.60 (0·40, 0·89)	0·012
Agreeableness	1·01 (0·68, 1·50)	0·951
Conscientiousness	0.97 (0·66, 1·42)	0·861
Emotional stability	0·88 (0·60, 1·32)	0·550
Intellect/openness	1·17 (0·80, 1·71)	0·428
Family self-harm	1·80 (0·80, 4.05)	0·157
Friend self-harm	1.08 (0·49, 2·39)	0·835
Life events	1.00 (0·72, 1·40)	0·999
Early adversity	1.01 (0·42, 2·46)	0·982
Body dissatisfaction	0·59 (0·28, 1·25)	0·172
Sleep problems		
Waking in the night	1.93 (0·86, 4·31)	0·109
Insufficient sleep	2.09 (0·99, 4.41)	0·001
**Psychiatric/mental health variables**		
Any DAWBA diagnosis	0·63 (0·13, 2·92)	0·553
Hopelessness	1·19 (0·65, 2·91)	0·401
Depressive symptoms	0.99 (0·71, 1·38)	0·937
Substance use		
Alcohol, heavy drinking	0·83 (0·37, 1·92)	0·668
Cannabis, at least occasional use	2·58 (1·12, 5·92)	0·026
Smoking, at least weekly	2.13 (0·78, 5·77)	0·139
illicit drug use (post year)	2·52 (1·15, 5·50)	0·020
Suicidal plans	2·15 (0·55, 8·46)	0·273
NSSH frequency		
Not in the last year	(reference)	0·718
1-5 times	1·06 (0·50, 2·24)	
6+ times	1·47 (0·58, 3·69)	
NSSH method used during most recent self-harm episode		
Cutting	(reference)	0·207
Other	0·39 (0·11, 1·38)	
More than one	0·53 (0·12, 2.37)	

NSSH: non-suicidal self-harm
